# Hepatoprotective Effect of Tea Composite Solid Beverage on Alcohol-Caused Rat Liver Injury

**DOI:** 10.3390/foods12224126

**Published:** 2023-11-14

**Authors:** Zheng Tang, Li Zhan, Ranran He, Yufei Zhou, Quanquan Tang, Zhonghua Liu, Sheng Zhang, Ailing Liu

**Affiliations:** 1 National Research Center of Engineering Technology for Utilization of Functional Ingredients from Botanicals, Hunan Agricultural University, Changsha 410128, China; a1054953611@gmail.com (Z.T.); hrr9580@163.com (R.H.); zhouyufeittea@stu.hunau.edu.cn (Y.Z.); larkin-liu@163.com (Z.L.); zhangsheng@hunau.edu.cn (S.Z.); 2 Key Laboratory of Tea Science of Ministry of Education, Hunan Agricultural University, Changsha 410128, China; 3 Collaborative Innovation Centre of Utilization of Functional Ingredients from Botanicals, Hunan Agricultural University, Changsha 410128, China; 4 College of Bioscience and Biotechnology, Hunan Agricultural University, Changsha 410128, China

**Keywords:** hepatoprotective effect, tea polyphenols, *Ampelopsis grossedentata* extract, cassia seed extract, alcohol-drinking rats

## Abstract

Alcoholic liver disease (ALD) remains a major cause of liver-related morbidity and mortality worldwide. Tea polyphenols (TPs) possess strong antioxidant activity; cassia seed extract (CSE) has the effect of brightening the eyes; and *Ampelopsis grossedentata* extract (AGE) has the function of protecting the liver. However, the synergistic hepatoprotective effect of TP, AGE and CSE as a joint formulation is unknown. This study aimed to investigate the role of a tea solid beverage, composed of TP, AGE and CSE, on chronic alcoholic liver injury in rats and its underlying mechanisms via the analysis of transcriptomics and gut microbiota. The histopathological findings revealed that the tea solid beverage could reduce the production of fat vacuoles and inflammatory cell infiltration. Additionally, the tea solid beverage was found to effectively relieve the increase in the AST (from 424.85 U/L to 180.17 U/L), ALT (from 139.95 U/L to 85.88 U/L) and LDH (from 21.16 U/L to 13.35 U/L) enzyme activities and the expression of the inflammatory factors TNF-α (from 394.02 pg/mL to 214.44 pg/mL) and IL-6 (from 208.46 pg/mL to 116.59 pg/mL) caused by alcohol consumption. Further, it significantly enhanced the GSH concentration (from 4.53 pg/mL to 8.08 pg/mL) and SOD activity (from 84.70 U/mL to 156.94 U/mL) and decreased the MDA (from 58.61 mmol/mL to 36.58 mmol/mL) and TG (from 7.07 mmol/L to 3.43 mmol/L)) concentrations in the liver of rats. The analysis and identification of transcriptomics showed that the tea solid beverage intervention primarily protected the liver of rats with chronic alcoholic injury by up-regulating the differential gene *Hmgcs1* in order to increase the synthesis of ketone bodies and by down-regulating the differential gene *Pfkfb1* for the purpose of decreasing the glucose metabolism. Additionally, it was found that the tea solid beverage could significantly change the composition of intestinal flora in drinking rats by regulating mineral absorption, the pathways of bile secretion, the adipocytokine signaling pathway and the peroxisome balance of the intestinal flora, in order to protect alcohol-drinking rats’ livers. In conclusion, the tea solid beverage, consisting of TP, AGE and CSE, is a functional drink that prevents ketone metabolism, glucose metabolism and microbiome disorders induced by alcohol intake.

## 1. Introduction

Alcoholic liver disease (ALD) poses a significant threat to human health, and its incidence in China currently stands at 5.15%, with a rising trend observed [1]. The Global Status Report on Alcohol states that over 40% of the population aged 15 or above consumes alcohol worldwide, and approximately 2 million people succumb to various alcohol-related illnesses annually [2]. China has the world’s highest number of alcohol-related mortalities, with up to 700,000 deaths per year [3]. At present, medical interventions for alcoholic liver disease primarily consist of pharmacological and surgical approaches, both of which are beneficial in terms of patient health. However, the extended use of chemical medications may increase the risk of harm to other organs and the digestive system [4], while surgical treatment is typically reserved for cases of severe liver disease with poor prognoses. Prolonged alcohol abuse is associated with symptoms of intestinal dysbiosis, increased intestinal permeability and even endotoxemia [5], and alterations in intestinal microbiology resulting from alcohol intake include bacterial overgrowth, the release of bacterial derivatives and an altered microbiota balance [6]. Therefore, it is important to establish means of preventing and alleviating the various pathologies caused by alcohol consumption and their impact on bodily health. Tea polyphenols (TPs) play a role in scavenging oxygen free radicals in the body, exhibiting anti-alcoholic liver fibrosis [7,8], anti-inflammatory and antioxidant effects [9,10], as well as providing anti-bacterial effects and supporting intestinal health, all contributing to the prevention of cardiovascular diseases [11,12]. *Ampelopsis grossedentata* is rich in flavonoids and induces many pharmacological effects, such as antioxidant, hypotensive, lipid-regulating and anti-liver cancer effects [13,14]. It may enhance the activity of antioxidant enzymes in the hepatic tissue, diminish the concentration of lipid peroxidation and inflammatory mediators and provide protective benefits to the liver [15]. Cassia seeds exhibit laxative, hypolipidemic, hepatoprotective and antimicrobial properties, making them an excellent source material for food and health products [16,17]. Furthermore, the active ingredients found in cassia seeds are known to protect against both acute and immune liver injuries [18]. Studies have shown that tea, *Ampelopsis grossedentata* extract (AGE) and cassia seed extract (CSE) possess antioxidant, anti-inflammatory and hepatoprotective effects and reduce lipid peroxidation. In China, cassia seeds are employed as dual-use resources for both medicine and food, additionally being used to brighten the eyes. Conversely, *Ampelopsis grossedentata* is a new food resource that affects liver protection. In previous studies, it has been demonstrated that the utilization of AGE and CSE may mitigate liver injury caused by alcohol consumption [19]. This study investigated the synergistic effects of the target and mechanism of action of TP, AGE and CSE on liver protection, aiming to explore the protective effect and regulatory mechanism of three combinations of plant-based solid drinks on the liver and intestine of alcohol-fed rats. Our research employed a range of techniques, including cell testing, biomarker analysis, transcriptomics and intestinal flora analysis, utilizing each with the aim of providing a sounder theoretical foundation for the development and utilization of plant-based solid drinks, healthy food and traditional Chinese medicine.

## 2. Materials and Methods

### 2.1. Chemicals and Reagents

TPs with a purity of over 98% and CSE were supplied by Hunan Sunfull Bio-tech Co., Ltd. (Changsha, China) and quantified via UV. AGE was supplied by Hunan Sunfull Bio-tech Co., Ltd. (Changsha, China) and quantified via HPLC. Minimum essential medium (MEM) was purchased from Shanghai Zhong Qiao Xin Zhou Bio-tech Co., Ltd. (Shanghai, China). Fetal bovine serum (FBS) was purchased from Cyagen Biosciences Inc. (Guangzhou, China). Ethylenediaminetetraacetic acid (EDTA), 100 units per mL penicillin and 100 mg mL^−1^ streptomycin (p/s) were purchased from Beijing Coolaber Technology Co., Ltd. (Beijing, China). ELISA kits, including those for aspartate aminotransferase (AST), alanine aminotransferase (ALT), lactate dehydrogenase (LDH), interleukin-6 (IL-6), tumor necrosis factor-α (TNF-α), total glyceride (TG), superoxide dismutase (SOD), malondialdehyde (MDA) and glutathione (GSH), were purchased from Changsha Aoji Bio-tech Co., Ltd. (Changsha, China). RNA-easy Isolation Reagent, the HiScript II Q RT SuperMix for qPCR (+gDNA wiper) and SYBR Green Premix Pro Taq HS qPCR Kit were purchased from Vazyme Bio-tech Co., Ltd. (Nanjing, China). A Steady Pure Universal RNA Extraction kit was purchased from Accurate Bio-tech Co., Ltd. (Changsha, China). All other chemicals were of analytical grade.

### 2.2. Cell Experiments

#### 2.2.1. Cell Culture

Procell Life Science & Technology Co., Ltd. (Wuhan, China) supplied the Buffalo Rat Liver-3A cell line (BRL3A), which was cultivated in MEM with 10% FBS, 100 units per mL penicillin and 100 mg mL−1 streptomycin. All cells were incubated under conditions of 37 °C and 5% CO_2_. The medium was changed every other day in order to maintain cell growth. For subculturing, confluent cells were rinsed with PBS at the indicated times and then disaggregated in culture flasks using EDTA.

#### 2.2.2. Protective Effect of Tea Solids Beverage on Alcohol-Induced BRL3A Cells Injury

In this experiment, cell viability and cytotoxicity were determined using the CCK8 method.

After cell digestion and centrifugation, the recovered cells were placed in 1 mL of culture medium and blown well. Adding 10 µL to a cell counting plate, we then counted the cells. The number of cells in 1 mL of media was determined, and the cells were diluted as needed. Subsequently, they were allowed to cling to the wall for 24 h. Then, using a 2.5 mL syringe, the supernatant was extracted after 24 h. A total of 700 μL of 98% TP, AGE and CSE was prepared as 10,000.00, 5000.00, 2500.00, 1250.00, 625.00, 312.50, 156.25, 78.13 and 39.06 μg/mL solutions and then added to the same volume of medium and mixed. The blank group contained only medium. Ethanol was diluted with a medium at an initial volume fraction ranging from 20% to 0.078%. Each well received 100 µL solution, nine concentrations, one blank and six parallel sets for each concentration. Subsequently, they were left to incubate for 24 h. After 24 h, the supernatant was removed, and the cells were cultured for 1.5–2 h with 90 µL of medium and 10 µL of CCK-8 reagent. The absorbance at 450 nm, corresponding to the activity of the multifunctional enzyme marker, was measured in order to assess the cell viability.

We determined the concentration range of alcohol damage and the safe mass concentration range of 98% TP, AGE and CSE. We set the ratio of composite solid beverage particles for the cytotoxicity test, and the effect of solid beverage on the cell survival rate of rat alcoholic liver cell model was analyzed. Equations (1) and (2) were employed to calculate the cell viability of the experimental and blank groups, respectively.

(1) $$\mathrm{RA}=(\frac{A1}{B}+\cdots +\frac{An}{B})/n\times 100\%$$

(2) $$\mathrm{RB}=(\frac{B1}{B}+\cdots +\frac{Bn}{B})/n\times 100\%$$

Here, RA is the cell survival rate of the test group (%), RB is the cell survival rate of the blank group (%), A1-n is the optical density value of the cells in the test group at 450 nm, B1-n is the optical density value of the cells in the blank group at 450 nm, B is the average optical density value of the blank group and n is the number of experimental parallels.

#### 2.2.3. Tea Solid Beverage Prevents Alcohol-Induced BRL3A Cell Injury via *Nrf2* and *HO-1* Genes Expression

In order to determine the volume of alcohol required to cause injury, an RNA extraction kit, reverse transcription kit and fluorescence quantitative PCR kit were used to detect the expression of *Nrf2* and *HO-1* in cells injured by different volumes of alcohol. The primer sequences can be seen in Table 1. According to the established alcohol volume, the effect of tea solid beverage particles on the viability of BRL3A cell was investigated.

### 2.3. Animals Experiments

#### 2.3.1. Animals and Treatments

In this study, forty Sprague Dawley (SD) rats weighing 200 ± 20 g were obtained from Hunan SJA Laboratory Animal Co., Ltd. (Changsha, China) with animal license number SCXK (Xiang) 2019–0004. The rats were housed in groups of eight per cage and provided with unrestricted access to food and water in a controlled environment with a light/dark cycle of 12 h (25 ± 3 °C at 50–60% relative humidity). All animal procedures were approved by the Ethics Committee of Hunan Agricultural University (Changsha, China), permission number HAU/BREC-2021095 (Changsha, Hunan, China).

**Figure 1:**
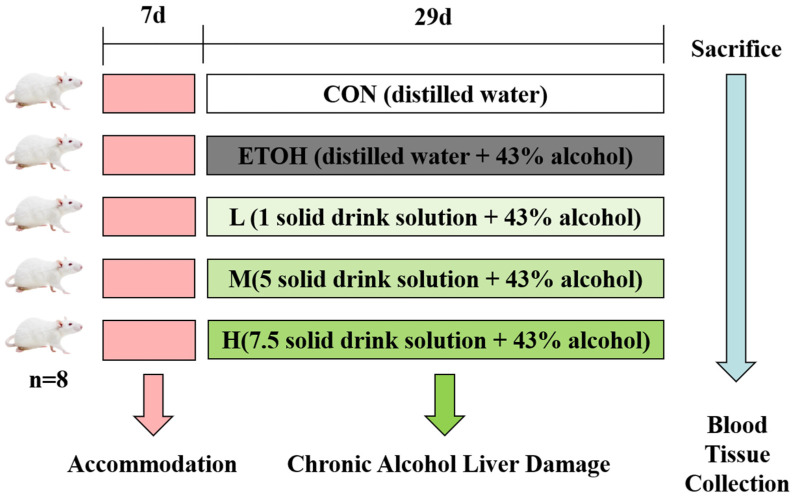
Scheme of the experimental design (n = 8 rats per group). Rats were divided into five groups: CON: control group; ETOH: ethyl alcohol model group; L: solid beverage low-dose group; M: solid beverage medium-dose group; H: solid beverage high-dose group.

The experimental design using animals is shown in Figure 1. After the end of adaptive feeding, the rats were randomized into five groups (n = 8): control group (CON), ethyl alcohol model group (ETOH), solid beverage low-dose group (L), solid beverage medium-dose group (M) and solid beverage high-dose group (H). The CON group received a daily intragastric administration of 7.5 mL/kg distilled water. The ETOH model group received a daily intragastric administration of 5 mL/kg distilled water, while the solid beverage groups received a daily intragastric administration of 5 mL/kg of solid beverage solution. One hour after undergoing gavage, rats from the ETOH model group and solid beverage group were gavaged with 2.5 mL/kg of 43° liquor on days 1 to 29 (L: 16.70 mg/kg 98% TP + 50.00 mg/kg AGE + 16.70 mg/kg CSE; M: 83.50 mg/kg 98% TP + 250.00 mg/kg CSE + 83.50 mg/kg CSE; and H: 125.25 mg/kg 98% TP + 375.00 mg/kg AGE + 125.25 mg/kg CSE).

At the end of the experiment, all rats were subjected to fasting for 16 h and anesthetized using chloral hydrate. Blood was collected from the abdominalis aortae and centrifuged at 3000 r/min for 15 min (4 °C). Serum was collected and stored at −80 °C. The liver and cecal contents were excised, immediately frozen in liquid nitrogen and stored at −80 °C until use. Portions of the livers were immersed in 4% paraformaldehyde fixative.

#### 2.3.2. Histopathology of the Liver

The fixed liver tissue was subjected to an alcohol gradient series for subsequent embedding in paraffin wax blocks prior to clearing in xylene and additional paraffin embedding. After dehydration, paraffin was used to embed the samples. Following dewaxing in xylene, hematoxylin and eosin (H&E) were applied to the sections in order to visualize histological changes in the liver.

#### 2.3.3. Biochemical Assays of the Serum and Liver Samples

Following the ELISA kit instructions, we measured the sera samples of ALT, AST, LDH, IL-6 and TNF-α and the liver samples of TG, SOD, MDA and GSH to determine concentrations.

#### 2.3.4. Transcriptome Analysis and q-PCR Verification

Liver RNA was extracted using the Steady Pure Universal RNA Extraction kit according to the instructions. The concentration and purity of RNA were measured with a Nano Drop micro-UV spectrophotometer (Thermo Fisher Scientific, Waltham, MA, USA), while RNA integrity and RIN value were assessed using the Agilent 2100 (Agilent Technologies, Santa Clara, CA, USA) and agarose gel electrophoresis, respectively. Employing magnetic beads with Oligo(dT) for A-T base pairing with ploy A, mRNA can be isolated from the total RNA and used to analyze transcriptome information. The RNA was isolated and fragmented, producing a 300 bp fragment that served as a template for the reverse transcription of single-stranded cDNA. Subsequently, double-stranded synthesis was performed to form a double-stranded structure, which was complemented with End-Repair Mix to form a flat end. Afterward, the “A” base was added to the 3’ end in order to connect to the Y-junction. The transcriptome genes were sequenced and correlated using the Illumina platform (San Diego, CA, USA), with sequence alignment and differential analysis carried out to confirm differential genes and identify relevant signaling pathways.

GO and KEGG analyses were performed using the Shanghai majorbio platform (Shanghai, China). Primer information for transcriptome gene expression verification is shown in Table 2.

#### 2.3.5. 16S rRNA Sequencing of Gut Microbiota

Extraction was carried out according to the instructions of the intestinal content DNA genome extraction kit, and the DNA concentration and purity were detected using a Nano Drop micro-UV spectrophotometer. The amplification primer sequence was used to amplify the 16S rRNA V3–V4 interval fragment. After recovering and purifying the amplified product, a sequencing library was prepared and 16S rRNA high-throughput sequencing was performed. Data analysis was performed when sequencing was finished. To create useful sequences, the original data were filtered and matched with the matching samples. The effective sequences were filtered and optimized, and chimeras were removed to obtain the optimized sequences. Then, the optimized sequence was divided into OTUs based on sample abundance. Subsequently, sample distribution, dilution curve and alpha diversity were evaluated, and Shannon analysis was performed. Samples were compared and grouped for beta diversity analysis, including PCA analysis, PCoA analysis and NMDS analysis, and we evaluated the bacterial community structure distinction between groups, assessed bacterial community abundance and finally performed a functional prediction of the bacterial community.

### 2.4. Statistical Analysis

GraphPad Prism 8.0. was used for statistical analysis and graphing, and the data were expressed as mean ± standard error of the mean (SEM). For all experiments, unless otherwise stated, the number of groups was n and n ≥ 6. The difference between the two groups was assessed via Student’s *t* test. Multiple comparisons were performed using one-way analysis of variance (ANOVA). *p* < 0.05 was considered statistically significant and *p* < 0.01 was considered statistically highly significant. *: compared with the blank control group, *p* < 0.05. **: compared with the blank control group, *p* < 0.01. #: compared with the ETOH model group, *p* < 0.05. ##: compared with the ETOH model group, *p* < 0.01.

## 3. Results

### 3.1. Effect of a Single Fraction on the Survival of BRL3A Cells

**Figure 2:**
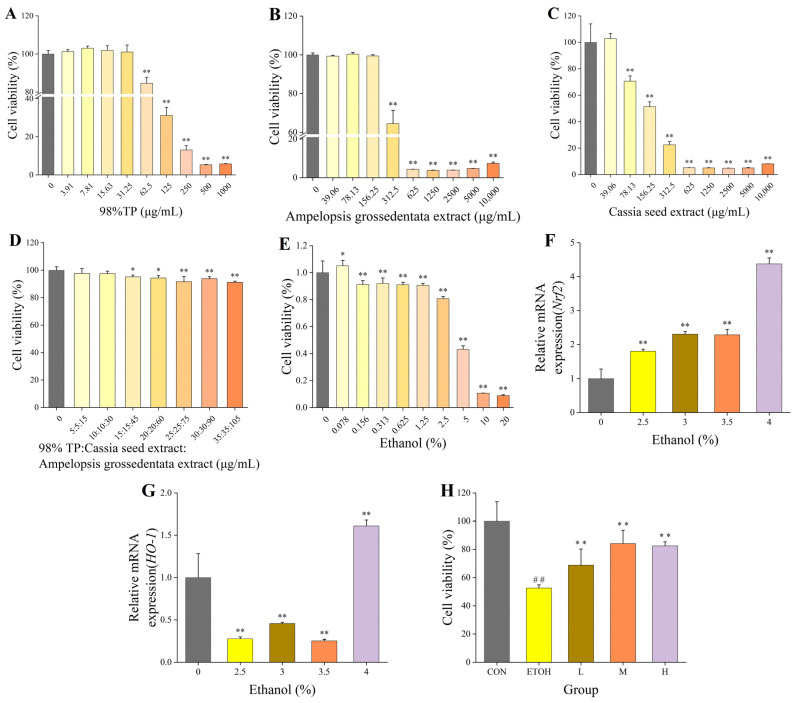
Cell test results. (**A**) Effect of 98%TP on cell viability of BRL3A; (**B**) effect of *Ampelopsis grossedentata* extract on cell viability of BRL3A; (**C**) effect of cassia seed extract on cell viability of BRL3A; (**D**) effect of composite solid drink on cell viability of BRL3A; (**E**) effect of ethanol concentration on cell viability of BRL3A; (**F**) effect of ethanol concentration on relative mRNA expression of *Nrf2*; (**G**) effect of ethanol concentration on relative mRNA expression of *HO-1*; (**H**) effect of 4% ethanol concentration on cell viability in the intervention of solid beverages. *: compared with the blank control group, *p* < 0.05. **: compared with the blank control group, *p* < 0.01. ##: compared with the ETOH model group, *p* < 0.01.

The BRL3A cells were treated with 98% TP, AGE and CSE, and the cell survival rate was measured after 24 h. At a 62.5 μg/mL concentration of 98% TP, the survival rate of the BRL3A cells was significantly reduced. Compared with the blank group, the difference was significant. Therefore, the safe concentration range of the 98% TP for the BRL3A cells was 0–31.25 μg/mL (Figure 2A). Similarly, the safe concentration range for the AGE and CSE was 0–156.25 μg/mL (Figure 2B) and 0–39.06 μg/mL (Figure 2C), respectively.

### 3.2. Effect of Tea Composite Solid Beverage on Cell Viability

The safe concentration range of the 98% TP, CSE and AGE was screened using a single-component cytotoxicity test. The maximum safe concentration of AGE was over three times that of the 98% TP and CSE. Based on these results, the optimal ratio of the tea composite solid beverage formula was determined to be 98% TP: CSE: AGE = 1:1:3. The concentration of the solid beverage was formulated within the safe concentration range of the single components. When the concentration of the composite solid beverage (μg/mL) was 15:15:45, there was a significant difference (*p* < 0.05) compared with the blank group, indicating that the cells were damaged, and the survival rate was reduced. As such, the safe concentration range of the composite solid beverage was 0–10:10:30 (Figure 2D).

### 3.3. Establishment of Alcoholic Liver Injury Model and the Effect of Tea Solid Beverages on Cell Viability

According to studies, when the cell survival rate is approximately 50%, the concentration of ethanol is suitable for damaging cells [20]. The highest value recorded was a 20% alcohol concentration. After 48 h of half-dilution damage, the cell survival was 80.7% at a 2.5% alcohol concentration and 43.2% at a 5% alcohol concentration, indicating that the suitable alcohol concentration for damage ranged from 2.5% to 5% (Figure 2E).

In order to determine the alcohol concentration of the establishment being modeled, we detected the relative expression levels of *Nrf2* and *HO-1* mRNA using a fluorescence quantitative PCR assay. Our results showed that, when the concentration was 4%, the relative mRNA expression levels of *Nrf2* (Figure 2F) and *HO-1* (Figure 2G) were significantly higher (*p* < 0.01). Therefore, we concluded that the concentration of the alcoholic liver injury model was 4%. Having identified the safe concentration range of the solid beverage and the concentration in the alcoholic liver injury model, we set up the control (CON), model (ETOH) and test (L, M and H) groups to determine the survival rate of the cells in the alcoholic liver injury model using CCK-8. The results showed (Figure 2H) that the cell survival rate of the ETOH group (52.49%) was significantly lower than that of the CON group (100%) (*p* < 0.01), indicating that the alcohol model was statistically significant. The cell survival rate of the L group (68.84%) was significantly lower than that of the CON group (*p* < 0.01) and significantly higher than that of the ETOH group (52.49%) (*p* < 0.05). The cell survival rate of the M (84.02%) and H groups (82.40%) was significantly different compared to that of the ETOH group (*p* < 0.01) and did not differ from that of the CON group (*p* > 0.05), indicating that the formulation of the M and H groups effectively alleviated the cell damage caused by alcohol.

### 3.4. Effect of Tea Composite Solid Beverage on the Liver Morphological Structure of Rats Drinking

**Figure 3:**
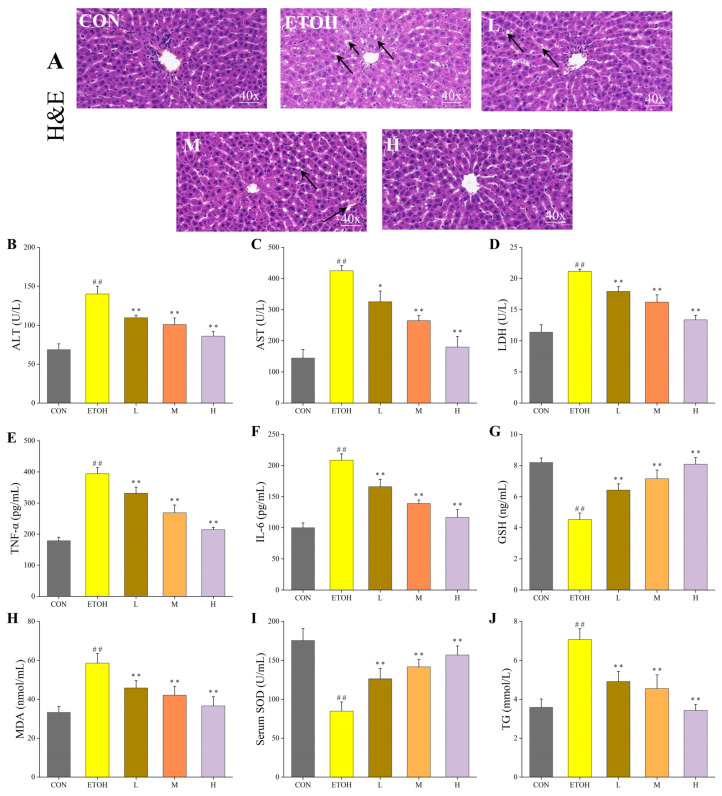
Effects of composite solid beverage on serum and liver-related indexes in drinking rats. (**A**) H&E staining of the rat liver (40×). (**B**) ALT. (**C**) AST. (**D**) LDH. (**E**) TNF-α. (**F**) IL-6. (**G**) GSH. (**H**) MDA. (**I**) SOD. (**J**) TG. *: compared with the blank control group, *p* < 0.05; **: compared with the blank control group, *p* < 0.01; ##: compared with the ETOH model group, *p* < 0.01.

The liver morphology of the drinking rats treated with the composite solid beverage was observed in Figure 3A. A morphological analysis of the livers of the rats receiving the composite solid drink revealed that the CON hepatocytes were arranged in a radial pattern surrounding the central vein. The hepatic cords demonstrated an orderly arrangement, without any indication of edema or steatosis. A small amount of cell death was observed. The hepatocytes in the ETOH group exhibited typical features, with apparent fat vacuole degeneration, uneven hepatocyte size, misaligned hepatic cords and ballooning degeneration. These features were accompanied by inflammatory cell infiltration and cell necrosis, indicating that alcohol by gavage caused damage to the morphology of the liver. In addition, the inflammatory cell infiltration and fat vacuolation in the L group were improved compared to the ETOH group. The liver cords in the M group were cleanly structured, exhibiting less fat vacuolation and inflammatory cell infiltration than in the low-dose group. The hepatocyte damage in group H was significantly improved, with neatly arranged hepatic cords and no fat vacuolation or ballooning degeneration, a situation that did not differ from that seen in the CON group. This suggested that the solid beverage could significantly mitigate the dose-dependent hepatocyte damage from alcohol.

### 3.5. Effect of Tea Composite Solid Beverage on Serum and Liver Injury-Related Indicators in Alcohol-Drinking Rats

Cardiac arterial blood supernatants were obtained and analyzed for AST, ALT, LDH, TNF-α and IL-6 (Figure 3B–F). Compared with the CON group, the activities of the ALT, AST and LDH and the concentration of the TNF-α and IL-6 in the ETOH group were significantly increased, indicating that the gavage dose was effective in damaging the liver. On the contrary, the solid beverage group displayed significantly lower activities of the ALT, AST and LDH and concentrations of the TNF-α and IL-6 than the ETOH group in a dose-dependent manner, indicating that solid beverages could reduce the secretion of inflammatory factors and the elevated activity of serum-related indicators caused by alcohol consumption, as well as maintaining normal liver metabolism and development. The GSH, MDA, SOD and TG of the rat liver were measured via ELISA (Figure 3G–J). The GSH concentration and SOD activity of the ETOH group were significantly decreased compared to those of the CON group (*p* < 0.01), while the treatment groups showed a significantly higher GSH concentration and SOD activity than the ETOH group (*p* < 0.05), indicating that the solid beverage could effectively alleviate the decrease in the hepatic GSH concentration and SOD activity caused by alcohol in a dose-dependent manner. Compared with the CON group, the MDA and TG concentrations were significantly higher in the ETOH group (*p* < 0.01). Conversely, the MDA and TG concentrations in the solid beverage group were significantly lower than those in the ETOH group (*p* < 0.01), and there was no significant difference between the solid beverage group and CON group, indicating that the solid beverage group effectively reduced the alcohol-induced increase in the MDA and TG concentrations. The above results showed that the solid beverage could effectively increase the GSH concentration and SOD activities to improve the body’s antioxidant capacity, reduce the MDA concentration to inhibit lipid peroxidation and reduce the TG concentration to inhibit fatty liver production, with the H group having a superior effect in alleviating liver damage. The change values of the physiological and biochemical indices are shown in Table 3.

### 3.6. Transcriptomic Analysis of Effect of tea Composite Solid Beverage on the Liver of Alcohol-Drinking Rats

**Figure 4:**
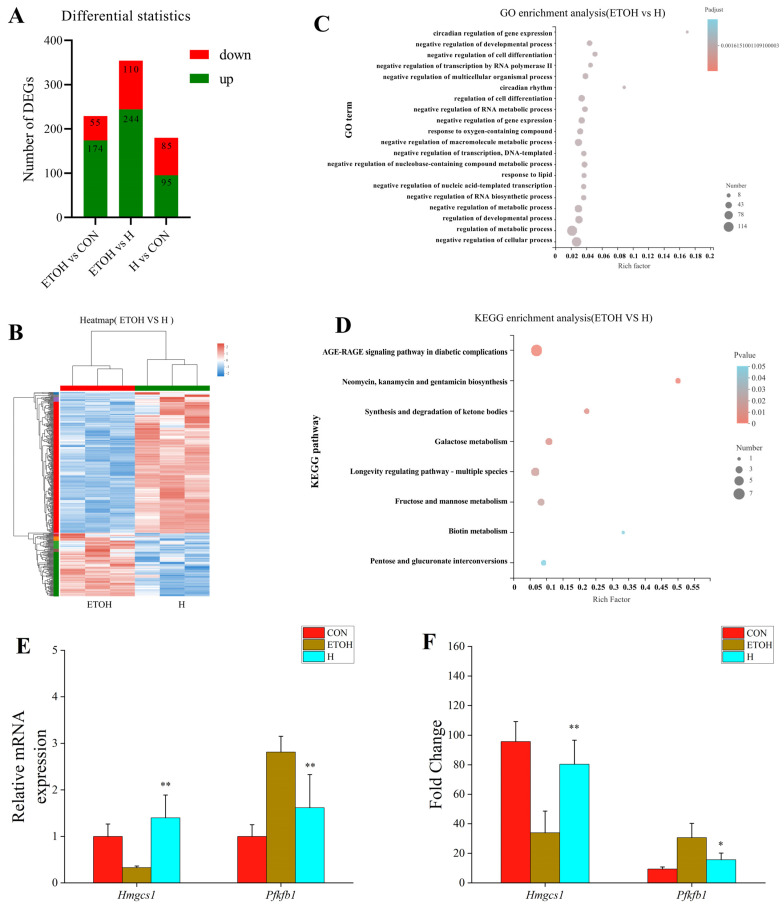
Transcriptome-related analysis of effects of composite solid beverage on liver of drinking rats. (**A**) Differential gene expression; (**B**) DEGs clustering heatmap; (**C**) GO enrichment analysis of DEGs in ETOH vs. H; (**D**) KEGG enrichment analysis of DEGs in ETOH vs. H; (**E**,**F**) QPCR verification results and DEGs identification. *: compared with the blank control group, *p* < 0.05; **: compared with the blank control group, *p* < 0.01.

Based on the above experimental results, the H group was more effective in terms of reducing liver damage. As such, RNA-seq was used to perform the transcriptomic analysis of the CON, ETOH and H groups. A differential analysis of the gene expression was carried out using the DESeq2 program. The results showed that there were 229 differential genes in the ETOH group compared with the CON group. These manifested as 174 significantly up-regulated genes and 55 significantly down-regulated genes. There were 354 differential genes in the ETOH group compared with the H group; 244 were significantly up-regulated genes, while 110 were significantly down-regulated genes. Additionally, the number of differential genes in the H group compared with the CON group was significantly lower than that in the ETOH group compared with the CON group, with 95 significantly up-regulated genes and 85 significantly down-regulated genes (Figure 4A). The intervention of tea solids in the H group reduced the differences between the alcoholic liver injury model rats and normal rats via the analysis of the differential genes in comparison to the CON, ETOH and H groups. According to the cluster analysis of 354 differentially expressed genes from the ETOH and H groups (Figure 4B), there were significant differences and more up-regulated differential genes in the H group than those in the ETOH group.

A GO enrichment analysis was performed on the differential genes in the ETOH and H comparison groups (Figure 4C). The findings indicate that genes with differential expression are primarily enriched in the domains of biological processes, molecular function, biological regulation, regulation of the biological process, regulation of the metabolic process, negative regulation of the biological process and regulation of the cellular metabolic process.

The KEGG enrichment analysis showed that the differential genes were primarily enriched in the pathways including neomycin, kanamycin and gentamicin biosynthesis, synthesis and degradation of ketone bodies, fructose and mannose metabolism, pentose and glucuronate interconversion, glucuronate interconversions, etc. (Figure 4D).

The ketone body synthesis and glycometabolism associated with the liver were screened from the KEGG enrichment pathways, and the enriched genes *Hmgcs1* and *Pfkfb1* were selected. Compared to the ETOH group, *Hmgcs1* was highly up-regulated in the H group, controlling the production of the ketone bodies, while *Pfkfb1* was dramatically down-regulated, controlling the gluconeogenic pathway (Figure 4E). The expressions of the differential genes *Hmgcs1* and *Pfkfb1* were confirmed via fluorescence quantitative PCR (Figure 4F). The results, obtained via qPCR, demonstrated that the gene expression of *Hmgcs1* and *Pfkfb1* was compatible with the transcriptome sequencing results. The transcriptomic results suggested that the composite solid beverage might protect against alcoholic liver injury in rats by regulating the metabolism and synthesis of lipids and sugars in the body.

### 3.7. Effect of Tea Composite Solid Beverage on the Intestinal Flora of Alcohol-Drinking Rats

Several studies have shown that gut microbes can influence the production and development of alcoholic liver injury. As such, 16S rRNA high-throughput sequencing was performed in order to further investigate the effects of the tea solid beverage on the intestinal flora of rats with chronic alcoholic liver injury.

**Figure 5:**
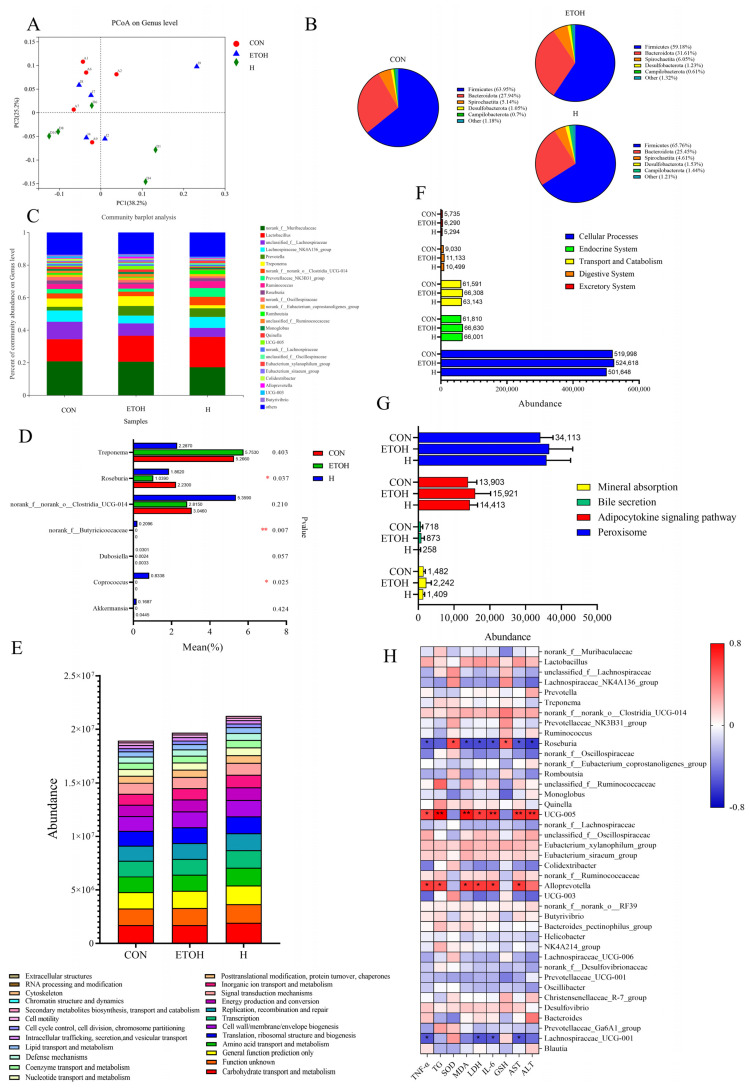
Effect of composite solid beverage on intestinal flora of drinking rats. (**A**) Beta diversity analysis; (**B**) community phylum composition distribution map; (**C**) the distribution map of the genus composition of the community; (**D**) multi-group comparison chart; (**E**) prediction of bacterial flora metabolic function based on KEGG pathway; (**F**) KEGG Pathway 2 Abundance Statistics Map; (**G**) KEGG Pathway 3 Abundance Statistics Map. (**H**) Spearman correlation analysis linking the abundance of specific genera in the intestinal flora of feces with serum and liver parameters. *: *p* < 0.05; **: *p* < 0.01.

The results of the PCoA analysis based on the Unweight Unifrac distance showed that the H group was on the opposite side of the axes and far away from the ETOH group (Figure 5A), while the first principal component PC1 was on the same side as the ETOH group but still far from it. They indicated that the CON group and the ETOH group were far apart. The difference in intestinal flora between the CON and ETOH groups suggested that the ingestion of solid drinks can change the structure of the intestinal flora caused by alcohol. The pie chart of the community abundance distribution, based on the phylum level of intestinal flora, showed that the top 5 most abundant common phyla in the CON, ETOH and H groups were Firmicutes, Bacteroidetes, Spirochaeta, Desulfobacterota and Campilobacterota. After the alcohol gavage, the ETOH group Firmicutes abundance (59.18%) decreased, and the Bacteroidetes (31.61%) and Spirochaeta (6.05%) abundance increased. Conversely, in the H group, the Firmicutes abundance (65.76%) increased, while the Bacteroidetes (25.45%) and Spirochaeta (4.61%) abundance decreased (Figure 5B).

The top 25 genera in the CON, ETOH and H groups were screened to perform a community composition analysis based on the genus level of the intestinal flora. Of the 141 genera in the three groups, the genera that accounted for the major components of each group were *norank_f__Muribaculaceae*, *Lactobacillus*, *unclassified_f__Lachnospiraceae, Lachnospiraceae_NK4A136_group*, *Prevotella*, *Treponema*, *norank_f__norank_o__Clostridia_UCG-014*, *Prevotellaceae_NK3B31_group*, *Ruminococcus*, *Roseburia*, *unclassified_f__Ruminococcaceae*, etc. (Figure 5C).

Compared to the CON group, *Roseburia* and *norank_f__norank_o__Clostridia_UCG-014* decreased in abundance in the ETOH group, and the uncontained genera *Akkermansia* and *Dubosiella* were in equal abundance in the CON and ETOH groups and increased in abundance in the H group. The *Treponema* abundance was higher in the ETOH group compared to the CON group. After the tea solid beverage intervention, the H group showed an increased abundance of *Ackermannia*, *Rhodobacter, norank_f__norank_o__Clostridia_UCG-014* and *Treponem* and a decreased abundance of dense spirochetes, while *Coprococcus* and *norank_f__Butyricoccaceae* showed enrichment in the H group. The species differences in abundance between the different groups of the microbial communities were screened for significant variation based on 160 genera in the H group and the association with liver disease (Figure 5D). A multi-species difference test revealed that, of the 160 genera, *Roseburia*, *Coprococcus* and *norank_f__Butyricicoccaceae* were considerably enriched in the H group, with the abundance values in that group significantly higher than those in the ETOH group.

The PICRUSt analysis of the intestinal flora of rats with alcoholic liver injury and tea solids beverage intervention was used to predict metabolic function. We performed the functional classification statistics based on the abundance of the OTUs (Figure 5E). The findings indicated that alcohol and solid beverage gavage had an impact on 24 functional pathways. The cell motility, the chromatin structure and dynamics and the cytoskeleton were the pathways by which the abundance decreased after alcohol administration and increased after solid beverage gavage, whereas RNA processing and modification experienced an increase after alcohol administration and a decrease after solid beverage gavage. Based on the OTU abundance, the KEGG Pathway Level 2 abundance statistics revealed that 39 pathways were altered, of which 5 pathways (Figure 5F)—including the cellular processes, endocrine system, transport and catabolism, digestive system and excretory system—showed an increase in abundance after alcohol gavage compared to the CON group and a decrease in abundance after the tea solid beverage intervention. An additional analysis of the third-order abundance of the 5 pathways identified 14 changed pathways, of which 4, viz., the mineral uptake, bile secretion, adipocytokine signaling pathway and peroxisome, were up-regulated in the liver following alcohol consumption and down-regulated after the tea solid beverage intervention. The results of the analysis of the intestinal flora of the rats in the CON, ETOH and H groups showed that the tea solid beverage intervention significantly improved the changes in the intestinal flora caused by long-term alcohol consumption, i.e., increased the abundance of beneficial bacteria and decreased the abundance of harmful bacteria; significantly regulated the abundance of *Roseburia*, *Coprococcus* and *norank_f__Butyricoccaceae*; normalized the abundance and diversity of the rat intestinal flora; and balanced the intestinal flora by regulating metabolic pathways, such as mineral absorption, bile secretion, the adipocyte factor signaling pathway and peroxisome, thus protecting against alcoholic liver injury in rats (Figure 5G).

Figure 5H shows the positive and negative correlations between the gut microbiota and serum/liver biochemical parameters. The levels of the parameters in the serum/liver were correlated with the abundance of several bacteria. For example, the pro-inflammatory factors TG, MDA, IL-6, AST and ALT in the liver showed a strong positive correlation with *UCG-005* (r = 0.80, r = 0.74, r = 0.66, r = 0.65 and r = 0.65, respectively), just as TNF-α and LDH were positively correlated with *UCG-005* (r = 0.57 and r = 0.61, respectively). TNF-α, MDA, LDH, IL-6, AST and ALT were negatively correlated with *Roseburia* (r = −0.53, r = −0.58, r=−0.57, r = −0.56, r = −0.54 and r = −0.63, respectively). SOD and GSH were positively correlated with *Roseburia* (r = 0.57 and r = 0.52, respectively), just as TNF-α, TG, MDA, LDH, IL-6 and AST were positively correlated with *Alloprevotella* (r = 0.61, r = 0.59, r=0.63, r = 0.62, r = 0.63 and r = 0.61, respectively). TNF-α, LDH, IL-6 and AST were negatively correlated with *Lachnospiraceae_UCG-001* (r = −0.53, r = −0.54, r = −0.52 and r = −0.54, respectively). 

**Figure 6:**
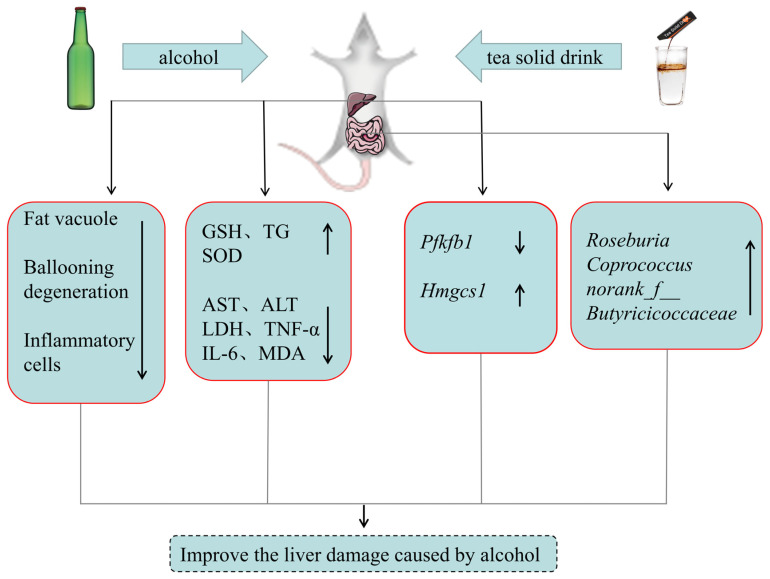
Mechanism of protective effects of composite solid beverages on the liver of drinking rats.

Figure 6 shows the mechanism of the protective effects of the composite solid beverages on the liver of drinking rats.

## 4. Discussion

ALD has become a common affliction that significantly impacts individuals’ daily lives [21]. Currently, interventions for alcohol-related liver disease, such as abstinence, surgery and medication, primarily target the intermediate and late stages of liver disease development. It is essential to intervene in or protect the liver from alcohol damage at an earlier stage, highlighting the need to explore new resources of functional beverages with the potential to improve people’s health [22]. The available evidence suggests that natural products such as TP, *Ampelopsis grossedentata* and cassia seed can protect the liver. TP can improve lipid deposition and oxidative stress in the chronic alcoholic liver by reducing the expression of the FAT/CD36 protein in hepatocyte membranes, intervening in ALD [23] and reducing intracellular ROS levels to alleviate stem cell steatosis [24]. *Ampelopsis grossedentata* is rich in flavonoids and has liver-protective and antioxidant properties [25]. The combination of cassia seed and hovenia dulcis thumb effectively improved the pathological changes in the liver tissue and liver function in rats with alcoholic fatty liver and reduced the expression of inflammatory factors [26]. However, the effect of a tea composite solid beverage formulated with tea, *Ampelopsis grossedentata* and cassia seeds on alcoholic liver disease is yet to be studied. Therefore, we investigated the hepatoprotective effects of this solid drink formulation and its potential mechanisms.

The ALD etiology is intricate. As common biochemical markers in liver function tests, serum ALT and AST activity are frequently utilized as biomarkers to assess hepatocyte damage and additionally display a positive correlation with liver function damage. Hepatocytes contain LDH, and hepatocyte injury promotes cell membrane permeability and LDH release [27]. TNF-α is involved in numerous physiological and pathological processes, such as immunity, anti-inflammation, anti-infection, anti-tumor, etc. TNF-α has potent anti-cancer properties and can cause cells to produce IL-6 and IL-1, as well as boost the expression of tumor necrosis factor [28]. The body strictly controls the expression and production of cytokines TNF-α and IL-6 in order to maintain appropriate levels and allow immunomodulatory functions to be carried out under normal circumstances. When foreign chemicals excite the body or cause lesions, the body either fails to develop an inflammatory response on time or produces an excessive amount of inflammatory response, which can seriously harm the body [29]. The research has demonstrated that ingesting solid beverages can effectively mitigate heightened levels of ALT, AST and LDH activity while reducing the elevated TNF-α and IL-6 levels that result from alcohol consumption. Xu et al. [30] showed that the gavage of TP in LPS model mice could increase the reduced SOD and GSH content in serum, reduce the MDA content, significantly reduce the content of inflammatory factors TNF-α and IL-1β in the liver and effectively improve the antioxidant and anti-inflammatory capacity of mice. Li et al. [31] showed through cellular and animal experiments that dihydromyricetin in *Ampelopsis grossedentata* can reduce AST, ALT and TG expression levels, attenuate alcohol-induced hepatic tissue steatosis, inhibit fatty acid synthesis and transport and promote fatty acid oxidative degradation. Pu et al.’s [32] study showed that the total anthraquinone from cassia seeds could reduce the expression levels of liver factors such as ALT, AST, IL-6 and TNF-α and inhibit the activation of the HMGB1/TLR4/NF-κB signaling pathway in LPS-induced acute liver injury rats, thereby reducing the inflammatory response. CSE can protect mice from acute alcoholic liver injury by inhibiting lipid peroxidation and reducing serum TG levels [33].

The liver tissue structure is the basis of normal function. Long-term alcohol consumption causes the disorganization of liver cords, the formation of fatty vacuoles and ballooning-like changes in the liver of rats. Conversely, the gavage of a liver protection solid drink will effectively alleviate the morphological and structural changes in the liver caused by alcohol consumption. Alcohol consumption worsens the liver’s ability to scavenge free radicals, diminishes the activity of antioxidants and endogenous antioxidant capacity and increases lipid peroxidation. These factors collectively contribute to the development of alcoholic liver injury. GSH can reduce toxic peroxides to non-toxic hydroxyl compounds capable of maintaining the body’s oxidative and antioxidant balance, and SOD can scavenge harmful superoxide anion radicals. These are important antioxidant enzymes in the body and can reflect the degree of oxidative damage in the body [34]. MDA, as the end product of free radical lipid peroxidation, can indirectly reflect the degree of the free radical attack in the body [35]. TG is one of the clinical diagnostic indicators of fatty liver. The liver breaks down fat into fatty acids and glycerol. Fatty acids can be turned into cholesterol, triglycerides and other compounds following lipidation in the liver cells [36]. Once fat enters the bloodstream, it can surpass the body’s natural threshold for fat storage, resulting in the accumulation of fat and ultimately leading to the development of a fatty liver [37]. Our results showed that solid beverage gavage could effectively alleviate the morphological and structural changes in the liver caused by alcohol consumption in a dose-dependent manner. A solid beverage could improve the antioxidant capacity of the body by increasing the GSH and SOD activity, reducing the MDA concentration, inhibiting free radical lipid peroxidation and reducing the TG concentration in order to limit the production of fatty liver.

After the alcohol gavage and tea solid beverage intervention in rats, the up-regulation of the differential gene *Hmgcs1* increased the synthesis of the ketone bodies, which acted as a regulator of mitochondrial function and reduced oxidative stress, while the down-regulation of the differential gene *Pfkfb1* reduced the glucose metabolism, oxidative stress and production of reactive oxygen species in the organism. *Hmgcs1* belongs to the group of HMG-CoA synthases, which are important intermediates in the synthesis of cholesterol and ketone bodies [38]. HMGCS converts acetyl coenzyme a into HMG-CoA, whereas HMGCL converts HMG-CoA into acetyl coenzyme a and the ketone molecule acetoacetate (AA), which can then be converted into D-beta-hydroxybutyrate (3HB) and acetone [39]. In the cytoplasm, SCFA can be converted into HMG-CoA by *Hmgcs1*, which carries out cholesterol biosynthesis through a series of cytoplasmic enzymes [40]. Ketone bodies are intermediary products of incomplete fatty acid oxidation in the liver, representing a unique form of energy transfer between the liver and extra-hepatic tissues [41]. Fluctuations in the concentration of ketone bodies reflect the state of the body’s sugar reserves. In general, ketone bodies are kept low in the body and the liver produces increased levels of ketone bodies as an energy source for extra-hepatic tissues when the glucose metabolism levels fall. The primary metabolic process of ketone bodies occurs in the mitochondria. Studies have shown that abnormal ketone body levels are associated with mitochondrial dysfunction and that these ketone bodies may play a role in controlling organismal health by regulating mitochondrial function, oxidative stress and signal transduction [42]. Mitochondrial DNA damage is higher in ALD patients than in healthy individuals, and reduced ATP synthesis leads to reduced respiratory chain complex activity, exacerbating mitochondrial dysfunction and reduced fatty acid β-oxidation, which in turn leads to reduced ketone body synthesis [43]. Numerous studies have shown that TP can significantly reduce mitochondrial permeability, alter pore openings and protect mitochondria from damage. TP also possesses the ability to inhibit and scavenge free radicals, providing protection to mitochondria against damage [44]. Polyphenols such as dihydromyricetin and prunetin, the active components of *Ampelopsis grossedentata*, can improve damage to the mitochondrial respiratory chain enzyme complex activity caused by hypoxia and raise the relative mitochondrial DNA content [45]. CSE can effectively improve mitochondrial dysfunction caused by a high-fat diet in mice, regulate insulin secretion and thus normalize glucose metabolism [46]. Lower levels of glucose metabolism are associated with higher levels of ketone metabolism in the body, and vice versa [42], and the two are dynamically and intimately related. Pfkfb is the main regulator of glycolysis, and *Pfkfb1* primarily exists in liver and skeletal muscle tissue [47]. The research has demonstrated that oxidative and carbonyl stress resulting from fructose and its metabolites, particularly glyoxal and methylglyoxal, plays a crucial role in the development of diabetic complications and chronic illnesses. A diet rich in bioactive compounds can inhibit this process. Various bioactive compounds and antioxidants, such as flavonoids and natural products like polyphenols, can slow down oxidative and carbonyl stress, and catechins and epicatechins protect against glyoxal or methylglyoxal-induced hepatocyte toxicity, inhibiting protein carbonylation and the production of reactive oxygen species [48]. When the body consumes too much sugar, there is a burden on the liver and intestine, and catechin extracts can effectively inhibit the transport of fructose and glucose [49]. Dong et al. found that the aqueous extract of cassia seeds could alleviate the chemical-induced increase in body mass, correct abnormal glucose tolerance, reduce blood fructosamine and insulin levels, correct lipid metabolism disorders and reduce hepatic lipid accumulation in rats [50]. In this experiment, the KEGG enrichment analysis of the differential genes showed that hepatic up-regulation of the differential gene *Hmgcs1* expression accelerated ketone body synthesis, whereas the down-regulation of the differential gene *Pfkfb1* expression slowed down the glucose metabolism after alcoholic gavage of the solid drinks in the rats. The research indicates a significant correlation between gastrointestinal microbiota and alcohol-related liver disease [51]. Diverse gut flora plays a crucial role in human health maintenance. When the balance of the human intestinal flora is upset, the species variety and abundance of the intestinal flora vary dramatically, resulting in inflammation, ageing, obesity, immunological dysregulation, metabolic abnormalities and tumor development [52]. The liver is the primary location for alcohol metabolism in the body, being responsible for secreting bile, controlling substance metabolism and maintaining the water–electrolyte balance. Long-term alcohol abuse can enhance the lipolytic effect of adipose tissue outside the liver cells, increase the free fatty acid content in the blood circulation, disrupt the oxidative balance in liver cells, inhibit fatty acid β-oxidation and induce lipid accumulation in the liver, implying that alcohol-related liver injury is closely associated with metabolic pathways [53,54]. Our findings showed that alcohol intake and tea solid beverage interventions can alter the structure and composition of the intestinal flora. Yan established a mouse alcohol model and evaluated the intestinal flora via continuous alcohol gavage. The findings revealed that the intestines of mice who consumed alcohol saw an overgrowth of bacteria, an increase in the Bacteroides and a decrease in the Firmicutes [55]. The decrease in the number of *Ruminococcidae* and increase in the level of Firmicutes and Bacteroides in the rumen resulted in chronic ethanol treatment in mice, according to other studies [56]. Bacteria in the human gastrointestinal tract are divided into four main phyla according to their natural properties, with the phylum Bacteroides, Firmicutes and Proteobacteria being the main dominant phyla [57]. Spirochetes are a type of bacteria, and one of their genera, Leptospira, can cause leptospirosis and other health problems [58]. The present study revealed that alcohol consumption resulted in a reduction in the Firmicutes population and an elevation in the populations of Bacteroidetes and Spirochaeta in the rat gut. However, treatment with the tea solid beverage effectively improved the decrease in the Firmicutes population while mitigating the overgrowth of Bacteroidetes and Spirochaeta caused by alcohol consumption. The intervention of the alcoholic gavage tea solid beverage increased the abundance of *Akkermansia*, *Roseburia*, *norank_f__norank_o__Clostridia_UCG-014* and *Dubosiella* while decreasing the abundance of *Treponema*, *Coprococcus* and *norank_f__Butyricicoccaceae* in group H. The genera significantly enriched in the H group via the analysis of variance were *Roseburia*, *Coprococcus* and *norank_f__Butyricicoccaceae*. *Roseburia* is a pivotal genus of intestinal bacteria that improves intestinal biodiversity; produces short-chain fatty acids, especially butyric acid; and possesses anti-inflammatory properties. Studies have shown that the administration of *Roseburia* in a mouse model of alcoholic fatty liver resulted in significant improvements in liver steatosis and inflammation [59]. Our results showed that the *Roseburia* levels increased after the tea solid beverage intervention, indicating that the tea solid beverage effectively improved the liver steatosis and inflammation in the rats. Group H was significantly enriched in *Coprococcus*, which is less abundant in the intestine and converts fructose into butyric acid [60], one of the short-chain fatty acids that enters the liver to participate in lipid and glycogen synthesis and also enhances mitochondrial function to accelerate fat oxidation [61]. *norank_f__Butyricoccaceae* is a genus of Firmicutes Clostridium, a beneficial bacterium that produces butyric acid to improve intestinal microbiology. Studies have shown that *norank_f__Butyricicoccaceae* in the intestinal flora of the experimental group supplemented with natural plant extracts was significantly richer than that of a standard group [62]. The considerable enrichment of *norank_f__Butyricoccaceae* in group H suggested that the tea solid beverage effectively improved the intestinal flora of the rats. Furthermore, the correlation analysis of the gut microbiota with liver injury parameters showed that an increased abundance of *Roseburia* and *Lachnospiraceae_UCG-001* resulted in decreased levels of the indicators of impaired liver function (LDH), hepatocytes subjected to apoptosis (AST) levels and inflammation (TNF-α and IL-6) levels. Therefore, tea solids can prevent alcohol-induced intestinal flora disorders and restore the indicators of impaired liver function, as well as hepatocytes subjected to apoptosis indicators and inflammatory parameters, to normal levels.

The functional prediction analysis of the intestinal flora showed that the pathways that altered the KEGG class III abundance after alcohol gavage and tea solid beverage interventions included the mineral uptake, bile secretion, adipocytokine signaling pathways and peroxisomes. Minerals are necessary for the human body through the dietary intake of minerals; indeed, excessive mineral absorption will cause an acid–base imbalance and affect the health of the body [63]. Adipokines have regulatory effects on glucose and lipid metabolism [64]. The peroxisome plays a crucial role in safeguarding cells against oxidative harm, regulating glycolipid metabolism and preserving inflammatory homeostasis within the body [65].

## 5. Conclusions

In summary, consumption of this tea solid beverage demonstrated beneficial impacts on hepatic fat vacuolation, inflammation, glucose metabolism and liver injury through the modulation of metabolic responses and microbial communities. The tea solid beverage intervention reduced the alcohol-induced elevations of the serum markers AST, ALT, LDH, TNF-α and IL-6; up-regulated the hepatic GSH concentrations and SOD activity; and down-regulated the MDA and TG concentrations. Tea solid beverages protect against liver injury by regulating the glucose metabolism associated with hepatic metabolic pathways, up-regulating the differential gene *Hmgcs1* to accelerate ketone body synthesis and degradation and down-regulating *Pfkfb1* to reduce glucose metabolism. In addition, the tea solid beverage improved the intestinal flora with an increased relative abundance of *Roseburia*, *Coprococcus* and *norank_f__Butyricicoccaceae*. However, this study only tested one ratio and did not determine the protective effects of other ratios or two-component pairs on rats with alcoholic liver injury. The efficacy of three-component/two-component pairs could be optimized through subsequent response surface testing. The present study was able to further analyze the role of the hepatic–intestinal axis in the regulation of alcoholic liver injury, and the histological data can be further analyzed and validated to investigate the specific links and mechanisms of action between the hepatic–intestinal axis.

This study investigated the protective effect of tea solid beverages on alcoholic liver injury. The results showed that the tea solid beverage has a liver-protective effect, which can provide the scientific basis for the production and market development of functional tea solid beverages. The above results showed that this tea composite solid beverage can protect the liver and serve as a valuable nutritional drink.

## Figures and Tables

**Table 1:** The primer sequence of *Nrf2* and *HO-1*.

Primers	Primer Sequence
*GAPDH*	Forward: ACAGCAACAGGGTGGTGGAC Reverse: TTTGAGGGTGCAGCGAACTT
*Nrf2*	Forward: GAGGATGGGAAACCTTACT Reverse: CTTCTTGCTCTTGGGAACA
*HO-1*	Forward: GTGCTCGCATGAACACTCTG Reverse: TGCAGAGGTAGTATCTTGAACC

**Table 2:** The primer sequence of *Pfkfb1* and *Hmgcs1*.

Gene	Primer Sequence
*GAPDH*	Forward: ACAGCAACAGGGTGGTGGAC Reverse: TTTGAGGGTGCAAACTT
*Pfkfb1*	Forward: CTTTCGCCCAGACAACACAGAGG Reverse: GCGGCTGAGATACTTATGGACATCC
*Hmgcs1*	Forward: CGGTTCCCTTGCTTCTGTTCTGG Reverse: CCTGGTGTGGCATCTTGTGTGAC

**Table 3:** Changes in physiological and biochemical indices in each group.

	CON	ETOH	L	M	H
ALT (U/L)	68.88 ± 6.61	139.95 ± 8.69	109.39 ± 3.11	100.89 ± 7.45	85.88 ± 5.46
AST (U/L)	145.00 ± 24.67	424.85 ± 14.15	324.71 ± 31.19	264.33 ± 14.80	180.17 ± 30.03
LDH (U/L)	11.41 ± 1.05	21.16 ± 0.30	17.91 ± 0.69	16.17 ± 1.07	13.35 ± 0.66
TNF-α (pg/mL)	179.30 ± 9.81	394.02 ± 17.33	331.56 ± 17.45	268.74 ± 22.36	214.44 ± 6.34
IL-6 (pg/mL)	100.13 ± 6.76	208.46 ± 8.80	165.76 ± 10.81	13.98 ± 5.12	116.59 ± 11.25
GSH (pg/mL)	8.20 ± 0.26	4.53 ± 0.38	6.41 ± 0.36	7.14 ± 0.50	8.08 ± 0.39
MDA (mmol/mL)	33.30 ± 2.70	58.61 ± 4.39	45.83 ± 3.38	42.15 ± 3.96	36.58 ± 4.21
SOD (U/mL)	175.40 ± 13.60	84.70 ± 10.57	126.23 ± 11.90	141.75 ± 8.43	156.94 ± 10.60
TG (mmol/L)	3.59 ± 0.38	7.07 ± 0.49	4.91 ± 0.47	4.56 ± 0.62	3.43 ± 0.28

## Data Availability

The data used to support the results of this study are available from the corresponding authors.
